# Telephonic Medical Toxicology Service in a Low-Resource Setting: Setup, Challenges, and Opportunities

**DOI:** 10.5811/westjem.2020.10.48534

**Published:** 2021-02-15

**Authors:** Eveline Hitti, Tharwat El Zahran, Hani Hamade, Brent W. Morgan, Ziad Kazzi

**Affiliations:** * American University of Beirut, Department of Emergency Medicine, Beirut, Lebanon; † Emory University, Department of Emergency Medicine, Atlanta, Georgia

## Abstract

Poisoning and envenomation are a global health problem for which the mortality burden is shouldered heavily by middle- and low-income countries that often lack poison prevention programs and medical toxicology expertise. Although telehealth or teleconsult services have been used to bridge the expertise gap between countries for multiple specialties, the use of medical toxicology teleconsult services across borders has been limited. We aim to describe the use of a United States-based medical toxicology teleconsult service to support patient care at a hospital in a middle-income country that lacks this expertise. This report outlines the logistics involved in setting up such a service, including the challenges and opportunities that emerged from establishing medical toxicology teleconsult service in a low-resource setting.

## INTRODUCTION

Poisonings and envenomations are an important global health problem. It is estimated that up to 75% or more of all poisoning-related deaths occur in low- and middle-income countries (LMIC).^1^ The lack of poisoning prevention programs and the scarcity of medical toxicologists have been recognized as contributing factors to poisoning in LMICs.^2^–^5^ Additionally, many of these countries lack the infrastructure for a national poison control center that would play a crucial role in guiding preventive measures.

While multiple studies have shown that patients managed by medical toxicologists have reduced hospital length of stay, hospital costs, and mortality compared with other patient groups where no medical toxicologist was involved,^6^–^7^ many countries lack medical toxicology training programs and expertise. A number of international collaborations have attempted to bridge medical toxicology education gaps in LMICs. These include teleconferencing networks through the Global Educational Toxicology Uniting Project (GETUP) program, supported by the American College of Medical Toxicology.^4^

Alternatively, teleconsultation has also been used in some countries to seek clinical expertise. Teleconsultation is defined as a “synchronous or asynchronous consultation using information and communication technology to omit geographical and functional distance.”^8^ It allows physicians in remote or low-resource areas to access specialist opinion that would otherwise be unavailable to them. Teleconsultation has been used in pediatrics, orthopedics, and general medicine to assist in decision-making, treatment plan, and referral.^8^ This platform of communication and delivery of care has also been increasingly used in international settings. One such example is the use of a toxicology teleconsultation, through an electronic mail system, by the US military and government healthcare providers serving overseas.^9^

While clinical teleconsultation is rising in popularity, the concept is not new to medical toxicologists through their roles at poison centers worldwide.^10^ For multiple decades, medical toxicologists have used poison center telephonic services as a means to provide expert input to the public and healthcare providers. In 2019, the World Health Organization registered 331 poison centers worldwide, with centers varying in their capacity and capabilities.^11^ However, this valuable resource is not available in all countries.

In Lebanon there is limited medical toxicology expertise and no national poison center to support providers with patient care. While Lebanon is classified as an upper-middle-income country, it faces many of the challenges of low-resource settings especially in healthcare: its healthcare system is fragmented with the majority of hospitals in the private sector, concentrated in urban areas, leaving more rural settings with limited access to quality care. Furthermore, the hospital accreditation process is limited to basic standard review leading to large variations in hospital capabilities and quality of service. Finally, 80% of the healthcare budget is spent on acute care primarily delivered in private hospitals, leaving the public sector under-resourced with no established national poison center.

In addition, the field of emergency medicine (EM) is newly developed with the first residency training program established in 2012 and, as of yet, there are no local or regional training programs for medical toxicologists. To address the limited access to medical toxicology specialists in Lebanon, the American University of Beirut Medical Center (AUBMC) initiated a telephonic medical toxicology service through a collaboration with the Medical Toxicology Section of the Department of Emergency Medicine at Emory University in Atlanta, GA. The clinical service team included an AUBMC toxicology officer with training in nursing and quality management who performed consultation follow-ups. Additionally, the team included international medical toxicology fellows who are non-US physicians with specialty training in EM, internal medicine, or pediatrics from their home country. These fellows are training in medical toxicology over a 24-month period at Emory University and function as the Certified Specialist in Poison Information within poison centers, handling the initial call and data collection, in addition to providing their toxicology recommendations after discussion with the medical toxicology physician on-call.

The telephonic service, established in 2015, has provided prompt clinical toxicology consultation for 669 patients to date. In this report we describe the initial set-up and service development, as well as challenges.

## DISCUSSION

### Conception and Telephonic Service Infrastructure

In 2015 a memorandum of understanding between AUBMC and Emory University was established whereby Emory medical toxicology faculty in the US would offer round-the-clock telephonic support for toxicology cases at AUBMC ED and medical center. AUBMC is a 358-bed tertiary care medical center, with an ED that receives approximately 55,000 visits and approximately 25,000 inpatient admissions annually. Pediatric patients comprise 20% of the ED visits and 17% of hospital admission. A medical toxicology faculty member at Emory University served as the medical director for the service, overseeing the call schedule and the database quality assurance process. While data entry occurred for quality assurance purposes, no formal consultation note by the medical toxicologists was documented in the chart. This resolved any medical liability issues for the medical toxicologist, while the quality assurance process ensured reliability and accountability of the telephonic advice.

**Population Health Research Capsule**

What do we already know about this issue?

Telehealth services have been used to bridge expertise gaps between countries for multiple specialties.

What was the research question?

We sought to describe the use of a US-based medical toxicology teleconsult service to support patient care at a hospital in a middle-income country.

What was the major finding of the study?

Our team successfully implemented a telehealth toxicology service; international collaboration is a viable opportunity.

How does this improve population health?

A medical toxicology teleconsult service can support patient care related to toxicology-related exposure, in a low-resource country.

Since this was not a telemedical consultation service but rather a telephonic resource, Joint Commission requirements for credentialing and privileging the medical toxicologists at AUBMC were not required.

### Communication

The utilization of the service was not mandatory and was made at the discretion of the treating physician. On-call toxicologist’s contact information was made available on AUBMC’s online scheduling program (Amion, Newtown, MA). The consultation request was handled by one of the three fellows at the Emory International Medical Toxicology Fellowship Program in Atlanta, GA, who collected data regarding the history, physical exam, and test results. The assessment and plan were finalized with one of the three medical toxicology supervising attendings.

Following the implementation of the electronic health record system (EPIC, Verona, WI) at AUBMC in November 2018, a toxicology consultation note with the toxicologist’s recommendations was uploaded into EPIC and signed by the on-site medical toxicologist who was ultimately recruited and assumed a third of the toxicology calls as part of a transition to in-house capacity. Language was not a barrier because the communication occurred in English, which is the default language used in all educational and professional activities at the AUB. On the other hand, occasional communication delays occurred because of the time difference. These were resolved through a back-up call system where attendings served as second call. There is usually a six- or seven-hour time difference given Atlanta’s location in the Eastern Standard Time zone.

### Utilization of Service

A total of 669 toxicological consultations have been received by the toxicology service to date. The majority of consults have been from providers at the AUBMC ED, inpatient services, and outpatient ambulatory clinics (92.7%, 1.5%, and 0.7%, respectively). Since the service was not publicized externally, only a few consults were from outside hospitals (3%) and few calls were from the public (2.1%). There was a 90% increase in the utilization of our service from 2015 (71 consults) to 2016 (135 consults), followed by a 21% increase in 2017 (164), and a slight decrease in 2018 (129 consults). A recent descriptive study of all teleconsult cases received between March 1, 2015–December 31, 2018, which included a total of 477 cases, reported the following main findings^12^: adult women and children less than five years old constituted a large portion of the cases; the majority of patients were found to have no effect or minor effects; only 20% resulted in moderate or major outcomes, and envenomation accounted for 3.8% of all the consults; intentional exposures were slightly more common than unintentional ones; almost half of the patients were treated and discharged from the ED and 34.2 % required admission; 49 patients (10.2%) left without completing care and were lost to follow-up, with the majority (N = 38) expected to have minor effects. The most common pharmaceutical agents involved were sedative/hypnotics/antipsychotics, analgesics, and antidepressants; the most common non-pharmaceutical agents involved were household cleaning substances, pesticides, bites, and envenomations. While benzodiazepine exposure was found to be common, opioid exposures were scarce in our population, which could be related to the strict government regulations on opioid prescribing and dispensing in Lebanon.

### Collaborations with Other Departments

The toxicology service initiated several internal collaborations to raise awareness about its capabilities and enhance its effectiveness. The leadership team held meetings with clinical departments, the clinical laboratory, and the clinical pharmacy department, the school of public health, the environmental core lab, and the zoology department. A policy was also established to properly handle hazardous specimens from the ED to the core lab in concordance with our occupational safety and risk management team. The nursing team was involved in all of the above procedures to ensure proper communication and standardize the care of toxicology patients.

### Poison Database and Quality Assurance

The medical toxicology fellows and the toxicology officer entered the cases into REDCap, a free, secure, web-based application designed to support data capture for research studies that is Health Insurance Portability and Accountability Act compliant The data included patient demographics, caller and hospital information, xenobiotic exposures, exposure route, history, physical exam findings, lab results, medical outcome, level of care, antidotes provided, and disposition. For the majority of variables, values were coded using a similar coding system adopted by the American Association of Poison Control Centers and used in its National Poison Data System.^13^ All patients were followed up by the toxicology officer, 24–48 hours post discharge, and all follow-up calls were documented in the database. For quality assurance, the database was frequently updated and monitored for data completion. The hospital used the database for quality control with oversight by the medical director. The service issued a monthly toxicology report to track the trends and patterns of exposure among the patient population.

### Toxicology Education

To bridge toxicology education gaps, multiple activities were provided to residents, medical students, and faculty members of various departments. These included monthly online webinars, journal clubs, and case discussions on common ingestions tailored to fit the poisoning patterns relevant to Lebanon. Additionally, seminars and grand rounds were provided to other departments to raise awareness about our service and introduce them to the common local toxicological exposures. Residents and fellows were also involved in preparing periodic, clinical case vignettes (ToxTidbits) that were circulated to all of the hospital’s healthcare physicians.

## LIMITATIONS and FUTURE OPPORTUNITIES

The provision of this telephonic toxicology resource faces several challenges and limitations. The inability to interview the patient directly potentially limits the ability of the consulting medical toxicologist to fully assess the patient. The consulting providers also encountered occasional difficulties with pharmaceutical products, local plants, snakes, and scorpions unique to Lebanon and poorly reported and characterized. Additionally, the consultant had to occasionally adjust the assessment and management recommendations to the locally available diagnostic modalities, laboratory tests, and antidotes. To address some of these limitations, consultants sought assistance from the AUB zoology and botany departments.

On the other hand, the service offered benefits and opportunities beyond its initial scope. The spectrum of exposures enriched the clinical experience of consulting physicians who do not frequently encounter in the US-specific exposures to chemicals like pesticides, hydrocarbons, and herbicides. This knowledge translated into several, peer-reviewed publications and case presentations. In addition, the initiative led to collaborations with the Ministry of Health including the development of the national health surveillance system for chemical exposures and multiple awareness campaigns on envenomations and poisonings captured through the service (pufferfish, toxic plants, and mushrooms).

Additionally, the service has also received consultations from several other Lebanese hospitals, emphasizing the need for such a resource at a national level. Lastly, this collaboration facilitated capacity building and trained one of the AUBMC EM residency graduates in medical toxicology at Emory University. This residency program was established in 2012 and is accredited by the Accreditation Council for Graduate Medical Education International.

## CONCLUSION

Our team was successful at implementing a telehealth toxicology service, and international collaboration is a viable opportunity. Future actions should ensure the sustainability and expansion of this resource nationally through local capacity building. This is best achieved through a partnership and formal collaboration between the Lebanese government, academic institutions, and hospitals.
